# Thermophysical Characteristics of Clay for Efficient Rammed Earth Wall Construction

**DOI:** 10.3390/ma16176015

**Published:** 2023-09-01

**Authors:** Cristian Petcu, Cornelia Florentina Dobrescu, Claudiu Sorin Dragomir, Adrian Alexandru Ciobanu, Adrian Victor Lăzărescu, Andreea Hegyi

**Affiliations:** 1 NIRD URBAN-INCERC Bucharest Branch, 266 Șoseaua Pantelimon, 021652 Bucharest, Romania; cristian.petcu@yahoo.com (C.P.); dragomirclaudiusorin@yahoo.com (C.S.D.); 2 Faculty of Engineering and Agronomy in Brăila, “Dunărea de Jos” University of Galati, 29 Calea Călarășilor, 810017 Brăila, Romania; 3 Faculty of Land Reclamation and Environmental Engineering, University of Agronomic Sciences and Veterinary Medicine of Bucharest, 59 Mărăști Boulevard, 011464 Bucharest, Romania; 4 NIRD URBAN-INCERC Iaşi Branch, 6 Anton Şesan Street, 700048 Iaşi, Romania; adrian.ciobanu@incd.ro; 5 NIRD URBAN-INCERC Cluj-Napoca Branch, 117 Calea Florești, 400524 Cluj-Napoca, Romania; adrian.lazarescu@incerc-cluj.ro; 6 Faculty of Materials and Environmental Engineering, Technical University of Cluj-Napoca, 103-105 Muncii Boulevard, 400641 Cluj-Napoca, Romania

**Keywords:** rammed earth walls, thermophysical performances, enthalpy, temperature buffer, moistude buffer

## Abstract

This case study focuses on twelve compacted clay soil samples to understand their fundamental physical and thermal properties. For each sample, the density, thermal conductivity, thermal diffusivity, specific heat, and drying shrinkage were assessed. The identification and characterisation of the materials were also carried out by positioning them into the ternary diagram based on the percentage of sand, silt, and clay. These properties are definitive for the performance characteristics of materials used in rammed earth wall construction. The aim is to provide information for better knowledge and prediction regarding the dynamic heat flow in rammed earth walls. Experimental results show a relatively wide range of values for each property, reflecting the diverse properties of the sampled clays. The thermophysical characteristics of the 12 types of earth analysed showed correlations with reports in the literature in terms of density (1490–2150 kg/m^3^), porosity (23.22–39.99%), specific heat capacity (701–999 J/kgK), and thermal conductivity (0.523–1.209 W/mK), which indicates them as materials suitable for use in the construction of rammed earth walls. Using test data, a dynamic assessment of heat flow through simulated rammed earth walls was performed. For a better understanding of the results obtained, they were compared with results obtained for simulations where the building element would be made of concrete, i.e., a mineral wool core composite. Thus, heat flux at the wall surface and mass flux, respectively, during the 16 years of operation showed similar evolution for all 12 types of clay material analysed, with small variations explained by differences in thermophysical characteristics specific to each type of S1–S12 earth. In the case of walls made from clay material, there is a stabilisation in the evolution of the water content phenomenon by the 5th year of simulation. This contrasts with walls made of concrete, where the characteristic water content appears to evolve continuously over the 16-year period. Therefore, it can be said that in the case of the construction elements of existing buildings, which have already gone through a sufficient period for the maturation of the materials in their construction elements, the rammed earth wall quickly develops a moisture buffer function. In the case of simulating a mineral wool core composite wall, it cannot perform as a temperature or humidity buffer, exhibiting an enthalpy exchange with indoor air that is only 4% of that of the rammed earth walls; consequently, it does not play a significant role in regulating indoor comfort conditions. Overall, there is confirmation of the temperature and moisture buffering capabilities of rammed earth walls during both warm and cold periods of the year, which is consistent with other reports in the literature. The findings of this research provide a better insight into clay as a material for rammed earth walls for more efficient design and construction, offering potential improvements regarding indoor comfort, energy efficiency, and sustainability. The data also provides useful information in the fields of architecture and civil engineering regarding the use of clay as an eco-friendly building material. The results emphasise the importance of thoroughly understanding the thermophysical properties of clay to ensure the efficiency of rammed earth construction.

## 1. Introduction

Rammed earth constructions, although having a history documented by evidence as far back as the Neolithic period, are still regarded with reluctance by some designers and builders as well as by some users [1,2]. Today, awareness of the need to reduce environmental impact, supported by current policies for sustainable development, is encouraging the construction industry to move towards the use of vernacular materials. Of these, earth, especially clay, is particularly important because it responds to the three main sustainability objectives: social (through its broad accessibility, providing housing—around 40% of the population lives in earthen houses [1], and creating jobs for various population groups), economic (through generally affordable costs), and environmental impact (both through its low polluting impact and its high reusability—recycling). The performance of rammed earth walls in terms of heat storage capacity, heat transfer, thermal resistance, etc. is of interest, especially in the context that, at the European level, it is estimated that more than 97% of buildings built before 2010 will require thermal rehabilitation works by 2050, given the need to reduce energy consumption to ensure indoor comfort [2]. Therefore, this trend towards eco-friendly building possibilities, combined with the need to rehabilitate existing buildings and renovate historical buildings (including some made of earth), requires a good knowledge of the advantages and disadvantages of earth-based building materials and elements, including an analysis of the mechanisms and possibilities to contribute to the regulation of air quality and indoor comfort with the lowest possible energy efforts. Therefore, rammed soil can be seen as a possible sustainable building material. However, standardisation in the field is often insufficient. From the point of view of timber frame construction, the most likely regulated field is on the Australian mainland, with three specific standards in New Zealand: NZS 4297, NZS 4298, and NZS 4299 [3,4,5] and the Australian Standards Association’s 2002 Australian Earth Building Handbook [6]. In Europe, the first standardised document was published in Germany from 1947–1956 and was withdrawn in 1970, and the national standard Lehmbau Regeln has been in existence since 1999. In Spain, the Ministry of Transportation and Public Works of Spain published a standardisation document for earth constructions, including rammed earth, in 1992. Other similar documents can be found in the USA, New Mexico’s New Mexico Building Code (1991), ASTM E2392/E2392M-10 [7], or Zimbabwe’s Standard Code of Practice for Rammed Earth Structures [8].

At the level of research reported in the literature, several results are currently presented that indicate the advantages of rammed earth constructions on indoor air quality through the possibility of regulating relative air humidity and temperature, with rammed earth walls acting as a moisture and heat buffer.

According to research, it is estimated that, in terms of thermophysical performance, a rammed earth block has a thermal conductivity coefficient of less than 0.7 W/mK in the dry state, according to Maniatidis and Walker [9], above 0.6 W/mK, varying with density and humidity, as presented by Losini et al. [10], or between 0.7860 and 0.846 W/mK, according to Suárez-Domínguez et al. [11]. Allam et al. report a thermal conductivity coefficient of 0.38–0.43 W/mK in the dry state and 0.55–0.62 W/mK in the wet state [12], and in contrast, Soudani et al. report a thermal conductivity coefficient in the range of 0.6–2.4 W/mK [13], which demonstrates the large variation in this parameter depending on several factors, of which humidity, density, and site-specific composition are defining elements. The literature reports also indicate a thermal transmittance of 1.9–2.0 W/m^2^K [9] and a specific heat of about 1832 J/kgK [1,14]. Additionally, in the literature, there are reports of specific heat capacities of 664 J/kgK according to Li et al. [1], 737 J/kgK according to Losini et al. [10], and 917 J/kgK according to Jiang et al. [15], in the range of 900–960 J/kgK confirmed by Cagnon et al. and Giuffrida et al. [14,16], or 750 J/kgK (dry state) and 1200 J/kgK (wet state) as presented by Allam et al. [12].

The soil thermal diffusivity (a [m^2^/s]) is the ratio of the thermal conductivity to the volumetric heat capacity and is an indicator of the rate at which a change in temperature will be transmitted through the soil by heat conduction. If thermal diffusivity is high, temperature changes are promptly transmitted through the soil. Soil thermal diffusivity is influenced by all the factors that influence thermal conductivity and heat capacity. However, thermal diffusivity is somewhat less sensitive to soil water content than thermal conductivity and volumetric heat capacity. The thermal diffusivity is a particularly useful parameter to aid in understanding and modelling soil temperatures [17,18].

Research focusing on the thermal improvement of rammed earth walls in Spain [19] provides a descriptive exploration into the thermal properties of various earth-based construction materials. The materials studied include, among others, rammed earth, sun-dried bricks, and compressed earth blocks (CEBs). Rammed earth, a traditional building material, has been tested at different densities, ranging from 1400 kg/m^3^ to 1900 kg/m^3^. The material with a density of 1400 kg/m^3^ had a value of 0.60 W/mK for thermal conductivity. As the density increases to 1600 kg/m^3^ and 1800 kg/m^3^, the thermal conductivity also increases to 0.80 W/mK and 1.00 W/mK, respectively. At the highest density studied, 1900 kg/m^3^, the thermal conductivity reaches 1.20 W/mK. Compressed earth blocks (CEBs), another common earth-based construction material, were also examined in the specialised literature. The densities of the CEBs studied ranged from 1700 kg/m^3^ to 1960 kg/m^3^. For the CEB, the thermal conductivity of the tested specimens does not show a consistent relationship with density, which proves that in this case the composition of the product is more important. For instance, a CEB with a density of 1700 kg/m^3^ reportedly had a thermal conductivity of 0.81 W/mK, while a denser CEB (1960 kg/m^3^) integrating recycled material had a lower thermal conductivity of 0.41 W/mK [19].

Although the characteristic values from a thermophysical point of view vary within wide limits and the thermal conductivity is generally high, it is worth mentioning that due to the thermal energy storage capacity, thickness, and massiveness of the walls, rammed earth provides a thermal inertia that allows for indoor thermal comfort with low energy consumption [20,21,22,23]. It is appreciated that, due to the technology of realisation, the earth building elements are initially characterised by high humidity, which can negatively influence the thermal and mechanical performance. This humidity, however, stabilises at an optimal equilibrium level after a longer period [12,24,25,26,27].

However, research reported by Hall et al. [28] showed that even this moisture content and time to equilibrium in rammed earth can be regulated by adjusting the particle size distribution of the clay-based composite.

The possibility of regulating indoor air humidity (RH) is another element of interest when discussing earth constructions. Research shows that factors that adversely affect the health of the population include bacteria, viruses, fungi, and mites, all of which thrive in conditions of high humidity. Maintaining RH in an optimal range of 40–60%, or at least keeping this parameter below 80%, can successfully contribute to minimising these indirect health effects [29,30,31]. In this context, some authors have proposed moisture buffering value (MBV) as a quantifiable indicator that represents the moisture storage/loss capacity, reporting values of 1.23–1.88 g/m^2^%RH, according to Liuzzi et al. [32] or 3.23–4.30 g/m^2^%RH, according to Niang et al. [24]. Another quantifiable indicator, the hygroscopic moisture storage, reported by Allison and Hall [33], is estimated to be in the range of 19.19–31.59 kg/m^3^. The demonstrated water vapour permeability of building elements made of clay soil, the specific capacity to store water in high humidity conditions and to release it in low RH conditions, provides direct benefits on the air quality of the inhabited space by regulating humidity and, consequently, on the quality of life [31,34,35,36,37,38,39,40]. Research indicates that the vapour diffusion resistance factor (μ) varies as a function of material density and compositional characteristics, with a maximum identified by Allison and Hall [33]. Li et al. [1] report a coefficient of 0.031 kg/(m^2^ s^0.5^) for untreated rammed earth to increase hydrophobicity and 0.0062 kg/(m^2^ s^0.5^) for similar samples but treated with a SILRES^®^ BS 1802 hydrophobizer.

Therefore, it is currently considered that some aspects defining the structure and characteristics of the material may influence the thermophysical performance of rammed earth walls. Some research reports a density for rammed earth of 1200–1900 kg/m^3^ [41], others indicate values of about 1800 kg/m^3^ [1], and others more than 2000 kg/m^3^ [10,14,15,42,43,44]. According to the literature, the soil suitable for rammed earth walls is estimated to be a balance of clay, silt, and sand proportions, with the minimum/maximum limits shown in Table 1. Overall, it is estimated that good rammed earth soil contains 5–35% clay, 30–80% sand, and 10–30% silt (mass percentage in relation to dry mass), with the caveat that in areas at risk of earthquakes, the amount of gravel should be reduced and natural fibres should be added to achieve dispersed reinforcement. If these limits are not met, solutions can be devised to allow for the use of additives in the composition [14,45,46,47]. As for plasticity, it is recommended to be in the range 10–25%, but preferably in the range 12–22%; density is recommended to be in the range 1700–2200 kg/m^3^ [9]; and soil moisture is used in the range 5–15% [10,14,48].

Therefore, it can be said that there is a large heterogeneity in the characteristics of clay earth as a building material, and these characteristics have a significant influence on the performance of rammed earth walls. Consequently, the aim of this work is to analyse the thermophysical performance of 12 types of clay soil extracted from Romania and to carry out a comparative analysis of the performance of rammed earth wall construction elements by simulating their behaviour in terms of energy and mass transfer capacity under predetermined conditions. The aim of this study was far from advocating the exclusive development of rammed earth walls at the expense of classical or modern techniques, which are largely based on reinforced concrete. However, it is not possible to ignore the existence of historical buildings, vernacular constructions, and modern constructions (from family houses to hotels and cultural centres, shopping centres, or museums) realised in rammed earth, as well as some of the advantages they present; therefore, it was considered important to underline these advantages. At the same time, as a motivation for the research approach carried out, it can be said that, although approached from different points of view, the performance of the constructions made of pounded earth is still far from being understood and fully quantifiable because the heterogeneity of the raw earth is very high; therefore, the behaviours can be very different from case to case. An approach such as the one in this study will contribute to the knowledge in the field, especially in the area of long-term behaviour analysis, as the chosen analysis method, the simulation of behaviour with the help of specialised software, will greatly contribute to the efficiency of the knowledge process (obtaining information in a much shorter time than in real-time tracking conditions) and, implicitly, to the increase in the degree of novelty.

## 2. Materials and Methods

### 2.1. Materials

**Figure 1:**
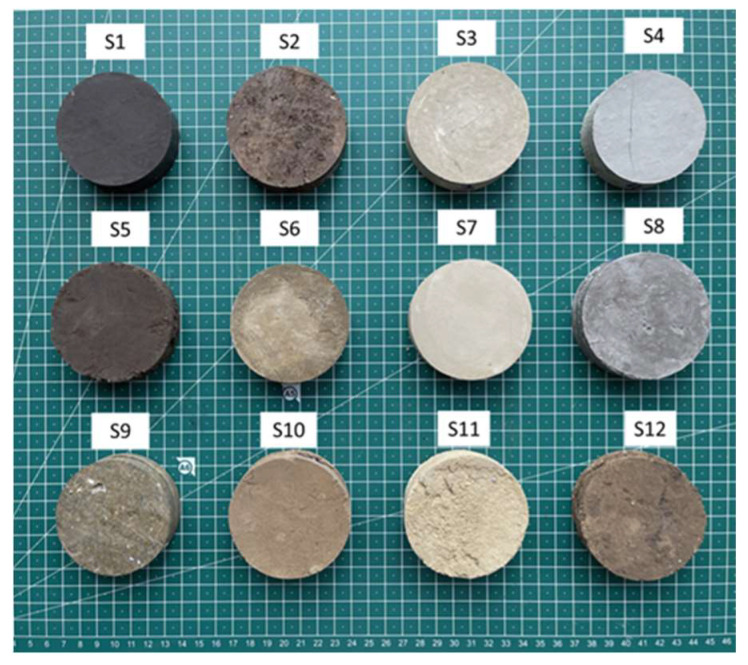
Appearance of the samples.

The experimental tests were carried out on twelve soil samples taken from different depths and geographical positions, all near the site of the ongoing construction activities. The sampling approach was designed to acquire a comprehensive representation of the soil profile in the vicinity of the work site, thereby providing valuable insights into the soil characteristics and evaluating the possibility of using them to construct rammed-earth walls. For easier identification, each soil sample has been individually coded as follows: S (soil) + extraction location number (1–12), resulting in the 12 identification codes of the 12 soil types analysed, i.e., S1–S12. Additionally, for the purpose of individual sample identification, Table 2 shows the extraction depths of each of the 12 soil samples analysed. The test samples were taken in 70 mm-diameter punches, as shown in Figure 1.

### 2.2. Material Analysis Methods

Analysis of the soil samples (S1–S12) was carried out to determine the physical characteristics (identification and condition), mechanical characteristics (compressibility), and thermophysical characteristics (thermal conductivity, thermal diffusivity, and volume heat capacity).

The identification of the soil types was carried out by determining the grain size distribution by the sedimentation method with a hydrometer according to the Romanian standard STAS 1913/5-85 [55]. The percentage distribution of grains by fraction was represented graphically by a grain size curve. Based on this curve and the ternary diagram representation, the classification of the soils according to SR EN ISO 14688-1 [56] and SR EN ISO 14688-2 [57] specifications was performed. As is well known in the literature, the ternary diagram is a very frequently used and useful tool both for soil classification and for other extended assessments (e.g., assessment of hydraulic properties or estimation of water management) [58,59,60,61].

The status was established by determining the limits of liquidity and plasticity. The plasticity characterisation of the soils was carried out according to the specifications of SR EN ISO 14688-2 [57]. Thus, plasticity is the property of cohesive or semi-cohesive soils between certain moisture limits, w_p_ − w_L_, to deform irreversibly under the action of external forces without volume variations or the appearance of discontinuities in their mass. Soils that have this property are called plastic soils (clays, powders), and soils that do not have this property are called non-plastic soils. The plasticity of soils extends over a range of moisture content limited by two moisture contents called plasticity limits (Atteberg limits): the upper plasticity limit, or liquid limit (w_L_), and the lower plasticity limit (w_p_). The range of moisture content variation for which soils behave plastically is called the plasticity index (I_p_) and is calculated according to Equation (1):

I_p_ = w_L_ − w_p_ (1)

To define the consistency state, the consistency index (I_c_) was defined according to Equation (2):

I_C_ = (w_L_ − w_p_)/I_p_ (2)

For each type of soil analysed, the activity in relation to water was evaluated, looking at the change in volume (swelling or shrinkage), as these indicators vary according to the particle size composition of the sample analysed. For the evaluation of the physical characteristics of soils with high swelling and shrinkage (PUCM), the laboratory determination methods provided by the Romanian standard STAS 1913/12-88 [62] were used, and the classification of the identified soils in terms of activity, quantified by the free swelling value (UL), was carried out according to NP 126: 2010 [63].

To determine the deformability characteristics, the soil samples were subjected to compressibility tests in an oedometer to obtain the oedometric deformation modulus (Eoed_200–300_) and the specific deformation at 200 kPa (ε_200_). The laboratory determination of the compressibility of the soils consisted of monitoring the settlement of the samples and their evolution over time under the effect of vertical axial loads applied in steps. Deformation measurements under constant loading were performed on cylindrical specimens with no lateral deformation and drainage on the lower and upper faces. The specimens were made from undisturbed soil samples collected as cores.

The set of special weights applied allowed for cumulative unit pressures (according to the specifications of STAS 8942/1-89 [64]) of 25 kPa, 50 kPa, 100 kPa, 200 kPa, and 300 kPa. Each load step was maintained until the tare weight was established (three successive readings at one-hour intervals that did not differ by more than 0.01 mm), after which the next load step was applied. During the determination, the final value of the tare (mm) was recorded for each loading step. After application of the last loading step and stabilisation of the settlement below it, the specimen was unloaded in steps up to the initial loading of 25 kPa. The assessment of the compressibility of soils based on the oedometric deformation modulus and specific compaction was carried out according to the specifications of the Romanian standard STAS 1243-88 [65] and the literature.

At the same time, all the soil samples were characterised by determining the density, expressed as the ratio between the constant mass of the sample and its volume. Based on the dry density and the density of the mineral skeleton, the porosity was determined.

The thermal performances were quantified using the ISOMET 2114 equipment (Manassas, VA, USA) and consisted of the determination of thermal conductivity [W/mK], thermal diffusivity [m^2^/s], and volume heat capacity [J/m^3^K]. The ISOMET 2114 is a portable measuring instrument designed for the direct measurement of the heat transfer properties of a wide range of isotropic materials, from soils to thermal insulation materials [66,67,68,69,70,71,72,73,74,75,76,77,78,79]. It is possible to equip two types of measurement probes: needle probes for soft materials and surface probes for hard materials. The ISOMET 2114 applies a dynamic measurement method based on a modified transient pulse method [80,81], which reduces the measurement time compared to steady-state measurement methods.

The procedures for conducting experiments to determine the thermophysical characteristics were executed on soil samples labelled S1–S12. Initially, these samples underwent a process of compression until they achieved a density equivalent to that of an earthen wall [82,83]. Following this, they were maintained in constant laboratory conditions until a state of constant mass was observed. The experimental tests were conducted under laboratory conditions, maintaining a temperature of 23 ± 2 °C and a relative air humidity (RH) of 50 ± 5%.

### 2.3. Analysis of the Thermophysical Performance of Rammed Earth Walls

The tests were carried out for each of the 12 soil types analysed (S1–S12). Given that the soil samples analysed were taken from Romania and vernacular constructions, specifically rammed earth constructions, are based on the principle of using local raw materials from deposits located as close as possible to the construction site, it was necessary to evaluate the behaviour of rammed earth walls for local climatic peculiarities. Romania’s climate is of a transitional temperate-continental type, marked in some areas by marine, continental, Scandinavian-Baltic, sub-Mediterranean, and Pontic climatic influences. Thus, in some areas, Mediterranean influences are more noticeable, with mild winters and a richer precipitation regime, while in other areas, Pontic influences are felt with rare but abundant rains. In the east of the country, continental influences are more pronounced; in the north, winters are frosty, and the climate is wetter and colder due to Scandinavian-Baltic influences; and in the west of the country, the influence of the Atlantic Ocean is felt, with moderate temperatures and relatively rich precipitation. Due to the varied relief, a climatic peculiarity is manifested within the Carpathian arc in Romania, with the area being cooler and characterised by high humidity throughout the year [84,85]. During the period 1961–2013, there was a trend of global warming that also manifested in Romania, especially through rising temperatures, relative stability of precipitation, with slight increases in the NW area and decreases in the Danube Delta area, and atmospheric humidity with mixed trends, decreasing in the N and SE and increasing in the southwestern area of the Carpathians [86]. Currently, the trend of global warming has intensified, and consequently, an increase in average annual temperatures is also recorded in Romania, with a tendency towards desertification in some areas. The multiannual average temperature varies from 8 °C in the northern area to over 11 °C in the southern area, and depending on the altitude, from −2.5 °C for the Omu Peak in the Bucegi Massif to 11.6 °C in the plains (Zimnicea, Teleorman county) [87]. Annual precipitation decreases from west (600 mm/year) to east (< 500 mm/year in the Eastern Plain of Romania, <450 mm/year in Dobrogea, and approximately 350 mm on the coast, while in mountainous regions, it reaches 1000–1500 mm) [87,88].

In building design, interior walls serve an important role as buffers, moderating the fluctuations of interior air temperature and relative humidity. This function is particularly important in the context of climate change [14,89,90,91,92,93], which has been shown to result in greater fluctuations in temperature and humidity [94,95,96,97,98,99,100,101]. This aspect is significant for maintaining thermal comfort in historical buildings [102,103,104,105] and for enhancing energy efficiency in new constructions [106,107,108,109,110,111].

To determine the hydrothermal performance of the interior wall in different cases of used materials, the WUFI^®^ 2D 4.2 simulation software is used. This software requires WAC files in order to have the necessary climate data for its calculations. However, the case study focuses on a building near the city of Constanța, Romania (44°09′ N, 28°36′ E), and the climate for this area is missing from our version of software. To overcome this issue, we used the online platform Shiny Weather Data [112] in order to process the ERA reanalysis dataset and provide us with a relevant WAC file for this location. This platform provides an efficient way to obtain the necessary WAC file by allowing users to select the desired area, the source for weather and solar data, and the time interval. The generated file contains:

- TA, the air temperature at 2 m above the ground, measured in °C [113,114];
- HREL, the relative air humidity, expressed as a value ranging from 0 to 1;
- ISGH [W/m^2^], the global solar irradiance on a horizontal plane (Irradiance, Solar Global Horizontal), which is the sum of the direct irradiation and diffuse irradiation and represents the total amount of shortwave radiation received from above by a surface horizontal to the ground [115,116,117];
- ISDH [W/m^2^], the direct component of irradiance, measured as incident on the horizontal surface [118,119];
- ISD [W/m^2^], the solar diffuse horizontal irradiance, which is the irradiance due to the diffuse solar radiation (scattered by the atmosphere), measured on a horizontal surface [119];
- ILAH [W/m^2^], the atmospheric counter radiation, usually known as downwelling longwave radiation, represents the amount of infrared radiation (longwave infrared) received from the atmosphere by a horizontal surface [120,121,122,123,124] as the greenhouse gases absorb longwave radiation from the Earth’s surface and re-emit a part of it to the surface, contributing to the greenhouse effect;
- CI, the cloud index, which is a measure of cloudiness or cloud cover, expressed as a value ranging from 0 to 1;
- RN, the rainfall, measured in millimetres;
- WD, the wind direction, measured in degrees, with North as 0°;
- WS, the wind speed, measured in kilometres per hour;
- PSTA, the atmospheric pressure at the station location, measured in hPa (hectopascals).

For this case study, weather and solar data provided by ERA5 were used. ERA5 is a global atmospheric reanalysis developed by the European Centre for Medium-Range Weather Forecasts (ECMWF) [125,126,127], which serves as the foundation for a wide range of research studies. These studies encompass various areas, for example, from climate change modelling [128,129,130], weather prediction [131,132], and hydrological processes [133,134], to urban heat island issues [135,136,137,138,139,140] and thermal comfort [120,141]. Reanalysis datasets are a scientific method for developing a comprehensive record of how weather and climate are changing over time. In producing reanalysis data, past observations are assimilated using a consistent modern analysis system [125,142] that generates datasets that describe the state of the atmospheric, land surface, and oceanic conditions over time.

As the focus is on discerning between different materials, it is important to use a relevant climate and to run the simulation over an extended period in order to negate the initial thermal and hygric conditions. For the simulation, the climate of the year 2022 is used, and the simulation is run for a total of 16 years. The material’s performance is evaluated using the last year’s data.

The rammed earth wall behaviour was analysed by simulation in the specialised WUFI^®^ 2D 4.2 software. This choice was made in accordance with other reports from the specialised literature that present results related to the hygrothermal behaviour of some construction elements obtained using this specialised software [143,144,145,146,147,148,149,150].

To determine hydrothermal performance, the following parameters were established:

- The wall was made from rammed earth, based on the characteristics of soil samples S1–S12. It is an interior partition wall with a thickness of 30 cm. For comparison, two other situations were considered, using materials frequently used in the construction of partition walls, namely, concrete (simulated using WUFI^®^ 2D 4.2 database data for concrete w/c = 0.5; density ρ 2300 [kg/m^3^]; specific heat c_p_ 850 [J/kgK]; thermal conductivity λ 1.60 [W/mK]; porosity 0.18 [%]; water vapour diffusion resistance factor μ 180 [-]) and a drywall partition system using gypsum board for faces (WUFI^®^ 2D 4.2 database data: ρ 730 [kg/m^3^]; c_p_ 850 [J/kgK]; λ 0.218 [W/mK]; porosity 0.65 [%]; μ 4.80 [-]) with interior mineral wool (WUFI^®^ 2D 4.2 database data: ρ 65 [kg/m^3^]; c_p_ 850 [J/kgK]; λ 0.032 [W/mK]; porosity 0.95 [%]; μ 1.1 [-]). In both scenarios (a concrete wall and a classic composite wall system with a mineral wool core), the total wall thickness was considered to be 30 cm. This was chosen to enable comparisons with the walls made from clayey soils that were analysed.
- Due to the fact that similar indoor climate conditions are considered on both sides of the interior wall, in WUFI^®^ 2D 4.2, a symmetry axis is used to minimise the number of elements and facilitate faster simulation. Therefore, the results are given for one side of the simulated wall.
- The interior conditions are derived by the software from the WAC climate file for Constanța, Romania, using the ASHRAE 160 climate type with heating only (i.e., no active cooling in the summer), a set point for heating of 20 °C, standard construction with two bedrooms, and a volume of 500 m^3^.
- Modelling the hygrothermal behaviour of the simulated element involves specific equations. WUFI^®^ 2D 4.2 uses two coupled equations, one for heat transport and the other for mass transport. These two coupled equations are solved simultaneously numerically using an implicit finite volume method of discretization [151,152,153]. For this study, we focused on analysing a field-area element without any thermal bridges. This allows for the one-dimensional formulation of heat (Equation (3)) and mass transfer equations (Equation (4)):
  (3) $$\frac{\partial H}{\partial \theta }\cdot \frac{\partial \theta }{\partial t}=\lambda \left(w\right)\cdot \frac{\partial^{2}\theta }{\partial x^{2}}+h_{v}\frac{\partial }{\partial x}\left[\delta_{p}\cdot \frac{\partial }{\partial x}\left(\varphi \cdot p_{v,\;\mathrm{sat}}\right)\right]$$
  (4) $$\frac{\partial w}{\partial t}=D_{\varphi }\frac{\partial^{2}\varphi }{\partial x^{2}}+\delta_{p}\frac{\partial }{\partial x}\left(\varphi \cdot p_{v,\;\mathrm{sat}}\right)$$
  where H is the total enthalpy [J], ϴ is the temperature [°C], t is time, λ(w) is the thermal conductivity coefficient [W/m·K], dependent on the specific moisture content w [kg/m^3^], h_v_ represents the enthalpy of vapourisation, δ_p_ is the water vapour permeability [kg/m·s·Pa], φ is the relative humidity [%], p_v,sat_ is the saturation vapour pressure [Pa], and D_φ_ is the liquid conduction coefficient [m^2^/s].
- For all the types of materials analysed, in the settings of the software used to simulate the behaviour of the wall in terms of energy and mass transfer, the period for which the simulation is carried out was set to 139,776 h (which would correspond to a time interval of 16 years (5824 days), as leap years were not taken into account in this period), with a constant discretization interval of one hour, resulting in evolution diagrams. This approximation, i.e., not taking into account the fact that every 3 years, the fourth year is a leap year, was considered to be possible and not to have a major influence on the results of the simulations because the volume of the recorded data sets is so large that missing 96 h (considering 4 leap years in the 16 years simulated) represents only 0.06% of the total 139,776 h. This total simulation period was subsequently divided into one-year periods to evaluate the existence of cyclicality in hygrothermal phenomena and depending on the specifics of the seasons (temperature, outdoor humidity, heating of the living space, indoor temperature, and humidity).
- To analyse the overall performance of interior walls made from various construction materials, the global parameter of enthalpy is integrated over the thermodynamic boundary using the data provided by WUFI^®^ 2D 4.2 for the last year of the simulation. This corresponds to a volume defined from the interface of the wall with the indoor air to the axis of symmetry of the wall, with an area of 1 m^2^ (Equation (5)):
  (5) $$H=\overset{t=8760}{\underset{t=1}{\sum }}\left|q\left(t\right)\right|+L_{v}\cdot \overset{t=8760}{\underset{t=1}{\sum }}\left|g\left(t\right)\right|$$
  where H represents total enthalpy exchange per square metre of wall surface [J/m^2^], the first term is the total heat flow through wall surface over the analysed yearly interval (both inside the thermodynamic volume and through the exterior), q(t) represents the heat flow integrated over one hour [Wh/m^2^], and the second term represents the latent component of enthalpy, with g(t) being the mass flow integrated over one hour [g/m^2^]. For the latent heat of water vaporisation (L_v_), a value of 2.257 kJ/g was used.

Enthalpy serves as a comprehensive indicator of the energy stored within architectural elements, encompassing various conditions. Traditionally, the focus has largely been on sensible enthalpy, which exclusively relates to temperature fluctuations in the building component, often excluding the broader term “enthalpy”. Nonetheless, our research using WUFI^®^ 2D 4.2 highlights a significant latent component (variations in the wall’s water content). Consequently, our analysis was expanded to encompass not just the variations in temperature (sensible enthalpy) but also the changes in moisture content (latent enthalpy). Sensible enthalpy refers to the change in the system’s energy as a result of a change in temperature. For a given amount of substance, its sensible enthalpy is proportional to its temperature change. In other words, sensible heat causes a temperature change without a phase change. Latent enthalpy refers to the energy absorbed or released during a phase change in a substance at a constant temperature and pressure. During a phase change, energy is absorbed or released, but the temperature remains constant.

Considering the type and location of the construction element for which the simulation was performed (an interior partition wall), as well as the fact that we were observing aspects related to energy transfer, such as functioning as a thermal buffer or humidity buffer, this analysis did not aim to obtain an evaluation from the perspective of the impact on overall consumption and the costs of achieving comfort conditions (heating or cooling costs). Therefore, it can be appreciated that aspects of the external climate had a lesser influence on the recorded results.

## 3. Results and Discussions

### 3.1. Soil Sample Characterisation

**Figure 2:**
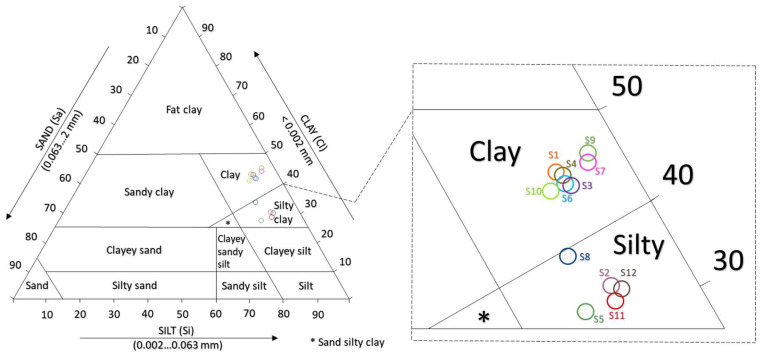
Representation of soil types on the ternary diagram.

For the analysed natural soils, the particle size classification was carried out by using the ternary diagram, which represents on the axes the granular fractions from SR EN ISO 14688-1 [56]. The results of the particle size determination showed that the analysed samples are delimited into two categories of soils: clay with a mainly clay fraction (S1, S3, S4, S6, S7, S9, and S10) and silty clay with a preponderant silty fraction (S2, S5, S8, S11, and S12), as illustrated in Figure 2.

From the point of view of physical parameters, the soils are characterised by medium plasticity and a medium free swelling index, and in terms of mechanical parameters, they have medium compressibility. The characterisation of the analysed soils S1–S12, according to plasticity and consistency status, plasticity limits, plasticity index, and consistency values, is presented in Table 3. The experimental tests to determine the UL free swelling showed that the analysed clay soils have an average activity in relation to water significantly changing their volume due to the variation in humidity and temperature (Table 4) and can be classified in the category of soils with high swelling and shrinkage according to NP 126-2010.

Experimental results for the determination of compressibility (deformability) characteristics led to the characterisation of these soils as having medium compressibility. The assessment of the compressibility of the soils based on the oedometric deformation modulus (Eoed_200–300_) and the specific heat (ε200) (Table 5) was carried out according to the Romanian standard STAS 1243-88 but also in accordance with the literature. At the same time, the experimental tests showed high heterogeneity in terms of thermal conductivity and specific heat, probably as a result of compositional and mineralogical differences. The thermophysical characteristics are shown in Table 5, and correlations with the literature reports were identified in terms of density [1,15,41], specific heat capacity [1,12,14,15,16], or thermal conductivity [9,10,11,12,13,19].

**Figure 3:**
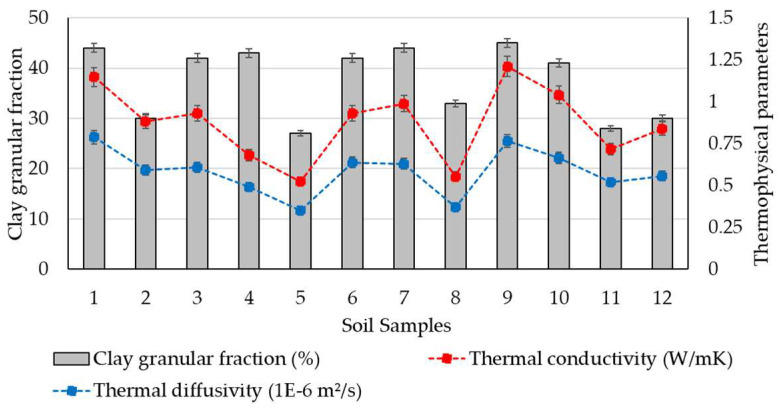
Variability of clay content with thermophysical parameters.

**Figure 4:**
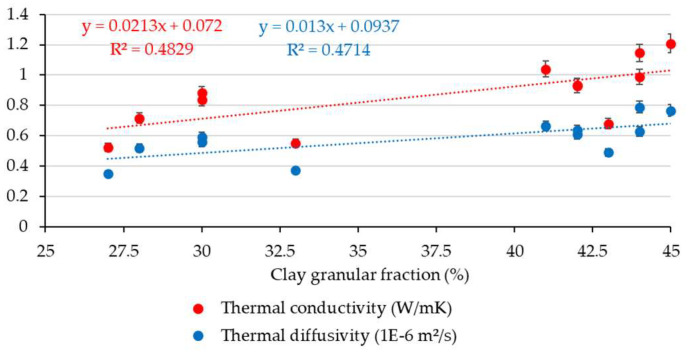
Simple correlation between clay content and thermophysical parameters.

**Figure 5:**
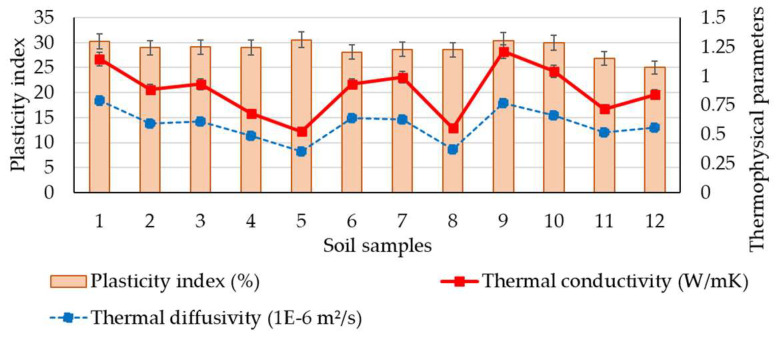
Variability of the plasticity index with thermophysical parameters.

**Figure 6:**
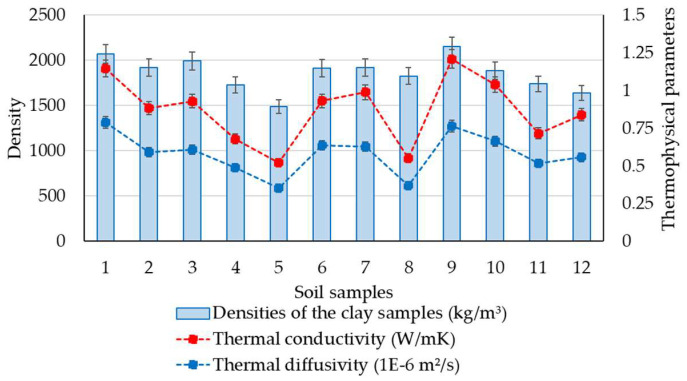
Variability of density with thermophysical parameters.

**Figure 7:**
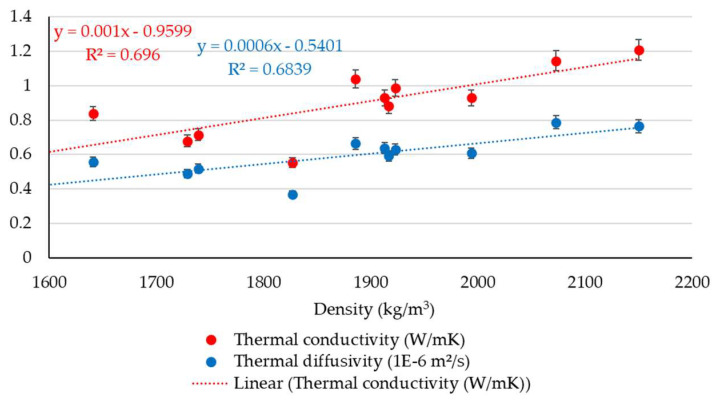
Simple correlation between density and thermophysical parameters.

Based on the results obtained from the measurements of thermophysical characteristics, a comparative synthetic assessment was carried out to estimate the existence or level of correlation with the soil geotechnical parameters. For this purpose, a series of data analyses have been performed by considering the clay content fraction, plasticity index, and density of the soils. The variability of thermal conductivity and diffusivity parameters with the clay content fraction is presented in Figure 3. It indicates a relative correlation between these parameters, as it was observed that an increase in clay can lead to an increase in thermophysical data, which reflected that the increase in clay fraction should be considered a potential variable for positive changes in thermophysical parameters. Moreover, a simple correlation analysis for this set of parameters was also performed. The results of the statistical analysis show moderately positive correlations between the selected sets of data, where the correlation coefficient (R^2^) is around 0.50, as shown in Figure 4, with similar trends for both thermophysical parameters. Although the possibility of identifying a potential correlation of thermal performances based on the clay fraction was examined, defined not by a linear function but by a polynomial, logarithmic, or other type of function, the correlation factor, R^2^, did not indicate a strong correlation in these situations. It consistently showed low values, suggesting that these parameters are strongly influenced by a variety of other variables. A similar approach was made by analysing the variability of thermophysical measurements with the plasticity index data, as seen in Figure 5. The results of the statistical analysis highlighted that there is no correlation between the analysed characteristics. The data analysis also showed that the thermal conductivity and thermal diffusivity generally increase with the increase in soil density, which suggests the potential for denser material with closer molecules to have a high thermal conductivity, as illustrated in Figure 6. Furthermore, extending this analysis of the data in terms of correlating geotechnical and thermophysical parameters, the specific heat capacity of soil samples is negatively correlated with density, whereas thermal conductivity is positively correlated with density. A moderate to slightly strong relationship was identified between density and thermophysical data, with a correlation coefficient (R^2^) around 0.70, as seen in Figure 7. While the correlation of these two sets of indicators—density to thermal conductivity and density to thermal diffusivity—is characterised by a better correlation index (R^2^) than when analysing correlation based on clay fraction (R^2^ > 0.6), it can also be appreciated that density is not the only decisive factor in the evolution of thermophysical indicators. Moreover, even changing the modelling function from linear to polynomial does not lead to a substantial improvement in the correlation index, which remains below 0.75.

Based on the results obtained from the experimental testing of the clay materials analysed, the following can be said:

- Characterisation tests of the 12 clay soils showed that they fall into the clay category (S1, S3, S4, S6, S7, S9, S10) and the silty clay category (S2, S5, S8, S11, S12), with medium water activity (free swelling, U_L_ 70–90%) and medium compressibility (10.26–16.72 MPa).
- The thermophysical characteristics of the 12 types of earth analysed showed correlations with reports in the literature in terms of density (1490–2150 kg/m^3^), porosity (23.22–39.99%), specific heat capacity (701–999 J/kgK), and thermal conductivity (0.523–1.209 W/mK), which indicates them as materials suitable for use in the construction of rammed earth walls.
- Overall, it can be said that there is a high degree of heterogeneity in terms of the thermophysical characteristics of these materials, and no mathematical correlation can be identified that indicates with sufficient precision (R^2^ > 0.9) the variation in one of these characteristics in relation to another, probably as a result of compositional, mineralogical, and grain size differences. In fact, this is the main difficulty encountered whenever the subject of rammed earth wall construction is discussed: the heterogeneity of the raw material and the need to characterise it from a thermophysical point of view.

### 3.2. Thermophysical Performance of a Rammed Earth Wall

Following the simulation using the specialised software WUFI^®^ 2D 4.2, the hygrothermal behaviour of rammed earth walls made from the twelve analysed clay materials, as well as two common materials (concrete and the drywall consisting of gypsum board and mineral wool core), was examined. The results obtained indicate the behaviour of the walls in several situations: the cold season, when heating the living space is required and a constant indoor temperature of 20 °C is imposed, and the warm season, when the living space is not artificially cooled, allowing the interior temperature to fluctuate naturally. Each case involves variations in the relative humidity of the air in the interior space.

**Figure 8:**
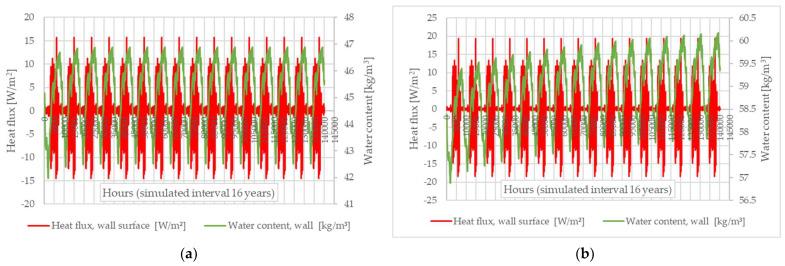
Values for the specific heat flow and water content, obtained over a simulation interval of 16 years: (**a**) rammed earth wall; (**b**) concrete wall.

Figure 8a illustrates the 16-year evolution of heat flux at the interface between the air and the wall surface, as well as the total water content within the thermodynamic contour (spanning from the wall surface to the wall’s symmetry axis). The results are those obtained using WUFI^®^ 2D 4.2 with the thermal characteristics of sample S1, as an example. However, similar patterns were observed for all the clay-based materials (S1–S12).

Figure 8b shows the evolution of a similar wall using concrete instead of clay compound. Interestingly, and somewhat in contrast to certain findings in the specialised literature [12,24,25,26,27], the clay-based wall shows a stabilisation in terms of water content evolution in the 5th year of simulation, while the concrete wall’s evolution appears to continue throughout the entire 16-year period. This observation is supported by a simple calculation: if the average water content, AWC (kg/m^3^), is calculated for each year of the simulation for the rammed earth wall, and then the percentage difference between each year’s AWC and the AWC characteristic for the 16th year of the simulation (relative to this 16th year) is determined, we find that the values decrease every year for the first 4 years, ranging from 0.84% in the first year to 0.01% in the 4th year, and then stabilise at 0% from the 5th year onwards. When a similar calculation is performed for the concrete wall, we find that the percentage difference in the annual AWC relative to the AWC value for the 16th year is also continuously decreasing, but the values fall within the range of 1.50% in the first year to 0.04% in the 15th year of the simulation. However, it should be specified that this apparent contradiction to the specialised literature may be because the simulation considered mature materials. It is known that the maturation of concrete is considered to occur 28 days after pouring, while the maturation of a rammed earth element occurs much more slowly and is even influenced by the manufacturing technology (water used in the preparation of the composition). For a concrete wall, the water is chemically integrated through a chemical reaction (hydration), and the result is the hardened gel-like matrix that integrates the different aggregates. That differs from the case of clay-based building elements, where water is needed to make the clay compound pliable during construction, but this water is not chemically bound and needs to be eliminated from the wall structure for it to harden and gain strength. Therefore, it can be said that, in the case of construction elements of existing buildings that have already undergone a sufficient maturation period, the rammed earth wall quickly assumes a buffering role with cyclical absorption–desorption intervals, the phenomenon being continuous and more stable than in the case of the concrete wall, which continues to evolve.

**Figure 9:**
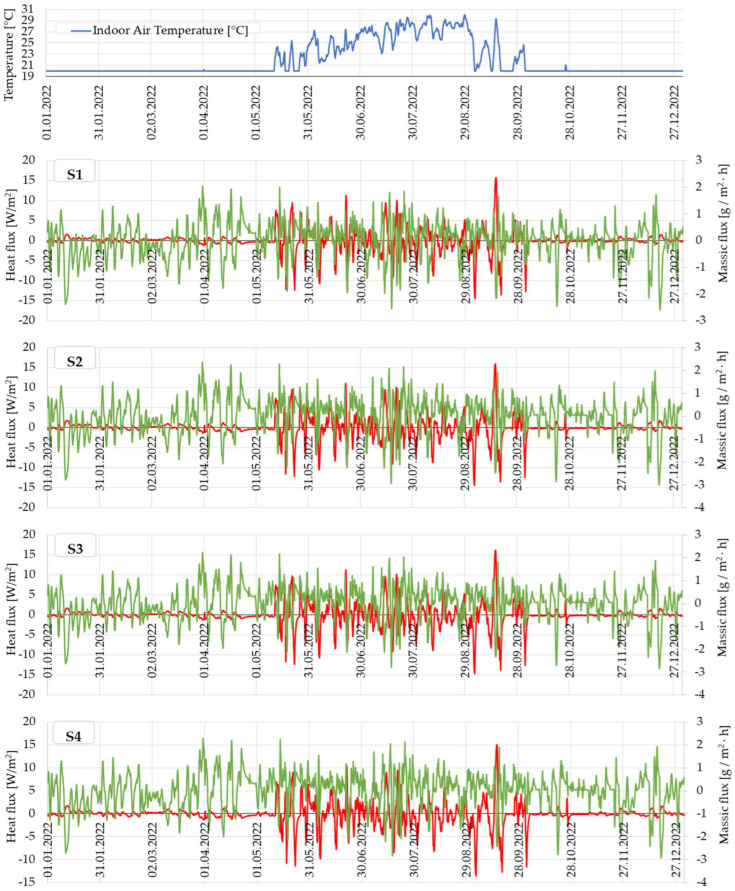

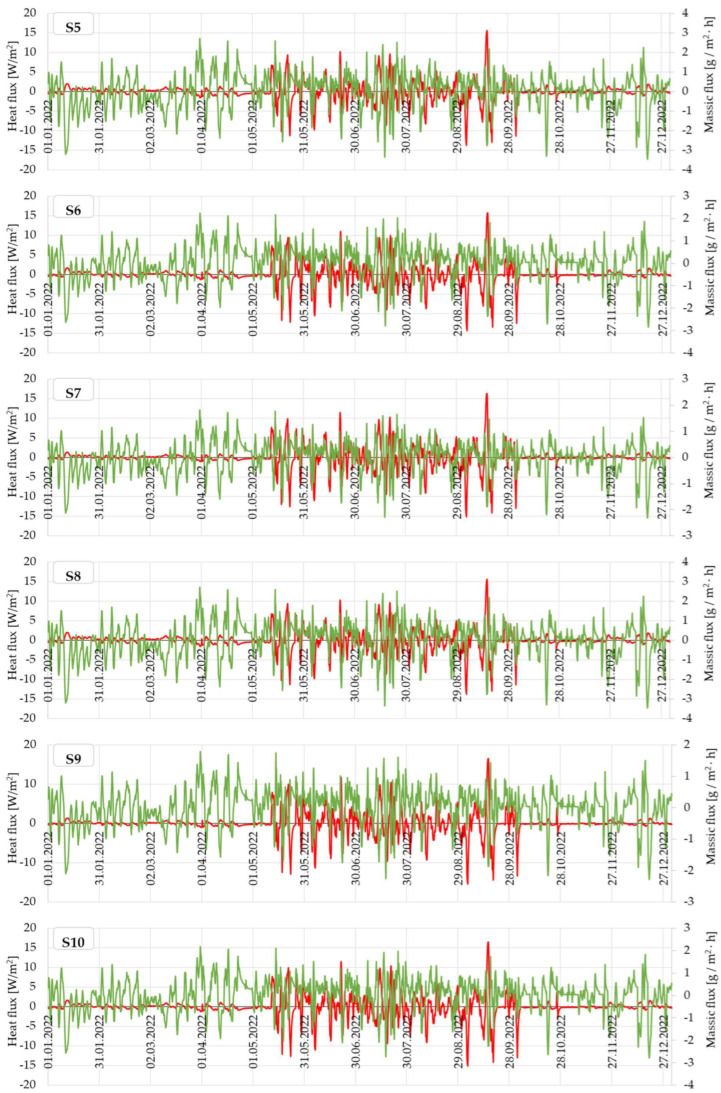

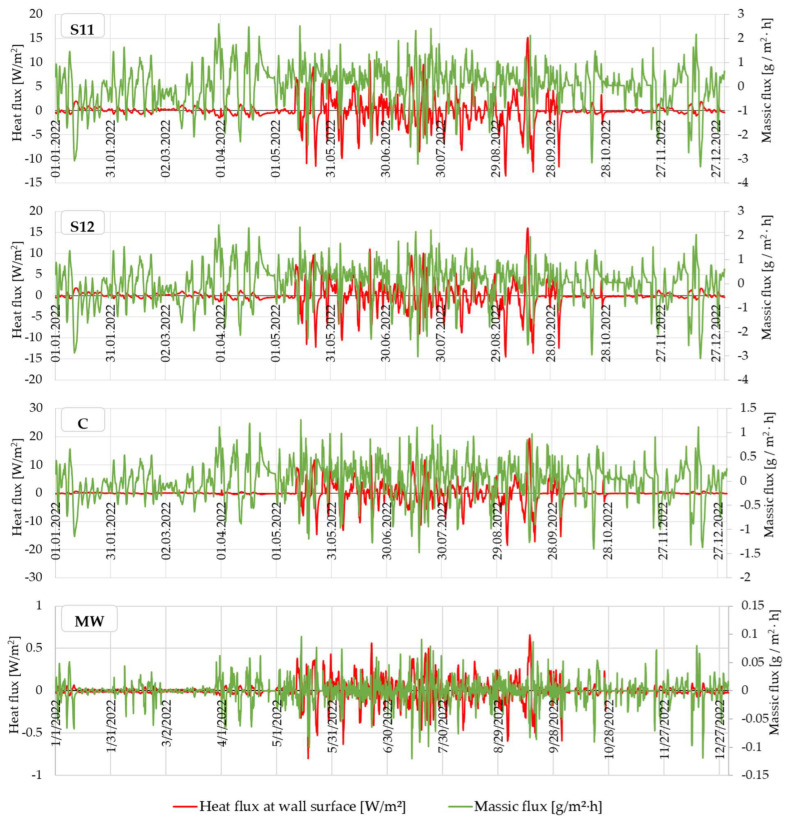
Evolution of the heat flux at the wall surface and the massic flux, respectively, for a thermodynamic contour between the face of the wall and its vertical axis of symmetry with a surface of 1 m^2^, over the last year of simulation, for a rammed earth wall made of clay materials (S1–S12), concrete (C), and a classic drywall with a mineral wool core (MW).

Refining the analysis period to the final year, the 16th, when essentially the entire process had reached a stable state, Figure 9 presents the records for all analysed materials. The heat transfer between the wall and the interior environment during winter is quite interesting, especially as the indoor temperature is consistently held at 20 °C. This fact can be attributed to the fluctuations in air humidity and the buffer effect created by the wall. Interestingly, this effect allows the enthalpic transfer of the interior wall to occur throughout the year, not just during the warm season as initially anticipated. Of course, during the warm season when the interior space is not air-conditioned, the interior wall experiences a higher degree of thermal stress and thermal flux. However, it is important to note that the transfer of heat and mass is a year-round process.

**Figure 10:**
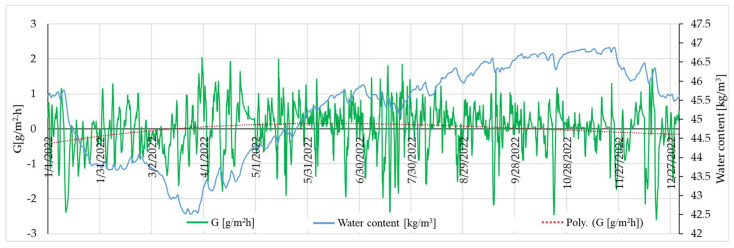
Evolution of mass flux and water content in the rammed earth wall (example presentation for S1) during the 16th simulation year.

Analysing the evolution of the mass flow (G) and the water content of the wall over the course of a year (from 1 January to 27 December of the 16th year of the simulated period), it was shown that the variation in the mass flow corresponds to a polynomial function that serves only as a wrapper (a synthesis) of the mass flow’s variation. This is exemplified by a rammed earth wall made from material S1 in Figure 10. This polynomial function intersects the *X*-axis at a certain point in March, transitioning from a domain of negative values to positive ones, therefore transitioning from an interval of drying (the wall has a negative derivative of water content with respect to time since the beginning of the year) to another interval in which there is an increase in the wall’s humidity. This occurrence, taking place in March, unfolds under the influence of increasing air humidity, which is in line with the general climatic evolution. This increase consequently leads to a rise in the humidity of the indoor air, prompting the wall to start accumulating moisture. During the summer, there are observable instances of momentary humidity fluctuations. For instance, on 17 July, the wall releases a significant amount of moisture as the relative humidity of the indoor air is quite low, but overall, the wall tends to accumulate moisture in the warm interval. The mass accumulation persists until around 10 October, when the envelope function intersects the *X*-axis once again. This time, it transitions into the domain of negative values, meaning that water exits the thermodynamical contour and vapours are released into the indoor air. This coincides with the seasonal drop in outside temperatures in the autumn and the initiation of the indoor heating system. It is also noted that during the winter, if the indoor air humidity significantly increases, some of this humidity is absorbed by the wall. In exchange, the wall releases a thermal flux towards the interior. For all other samples analysed, S2–S12, the general trend is similar. The only variation is the precise timing within the year when the *X*-axis intersects with the graphical representation of the envelope functions corresponding to each case. However, the months in which the two key events occur, namely March and October, remain consistent. This could serve as an indicator of the operational capacity of rammed earth walls as a buffer for controlling the temperature and humidity of an indoor space.

**Figure 11:**
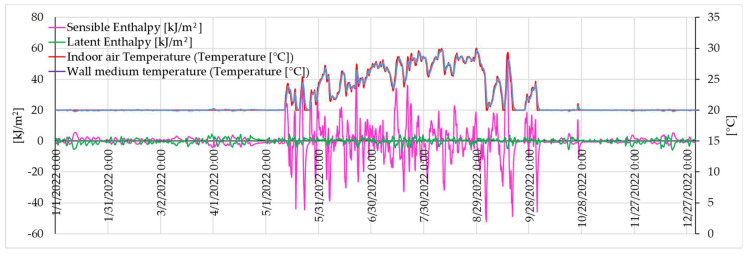
Enthalpy evolution of the rammed earth wall (example presentation for S1) during the 16th simulation year.

**Figure 12:**
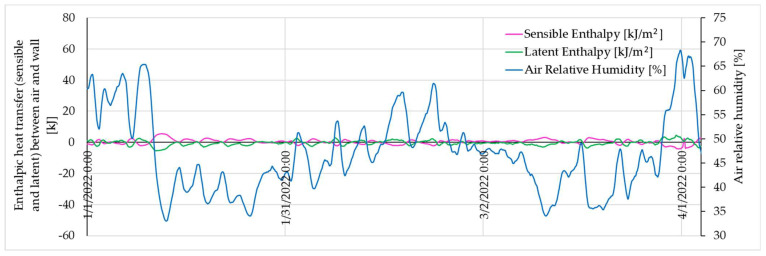
Detail of the enthalpy evolution of the rammed earth wall (example presentation for S1) over a short duration.

If the humidity significantly decreases, the wall releases moisture but absorbs the corresponding thermal flux, as can be observed in Figure 11 and Figure 12. It is important to specify here that the entire analysis is made from the perspective of mechanisms occurring at the wall level, with an indication of the influence on the characteristic parameters of the indoor air. As such, the sensible enthalpy provides information about the wall’s temperature, and the latent enthalpy offers insights into its moisture content. The information presented in Figure 12 further highlights that, in instances where the relative humidity of the indoor air exceeds the 50% threshold, the latent enthalpy reaches positive values. This indicates a mass transfer from the indoor air to the analysed volume, that is, towards the interior of the wall.

**Figure 13:**
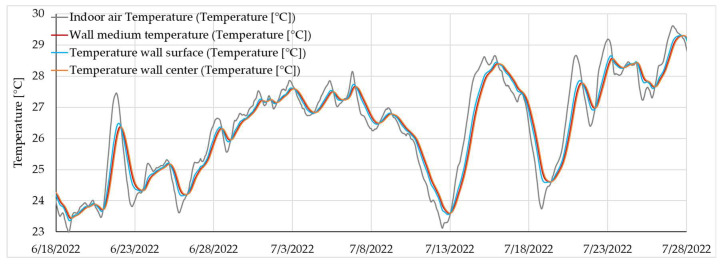
Detail of the rammed earth wall temperature evolution (example presentation for S1) over a short duration.

**Figure 14:**
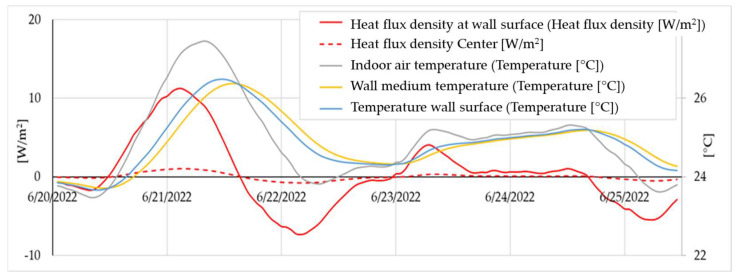
Detail of the heat flux evolution of the rammed earth wall (example presentation for S1) over a short duration.

Focusing even more on the analysed period, i.e., analysing the evolution of the hygrothermal parameters over a period of about one month, it can be consistently observed that there is a delay between the indoor air temperature, the wall surface temperature, the average wall temperature, and the temperature of the wall centre area (Figure 13). A similar delay is also observed for the variation in heat flux, indoor air temperature, wall surface temperature, and mean wall temperature (Figure 14).

**Figure 15:**
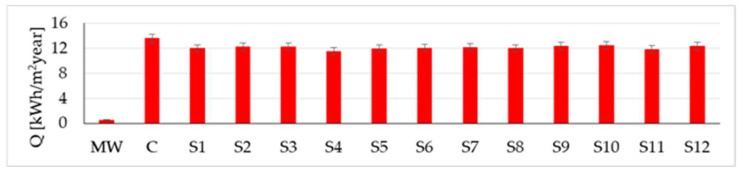
Heat flow Q integrated over one year.

**Figure 16:**
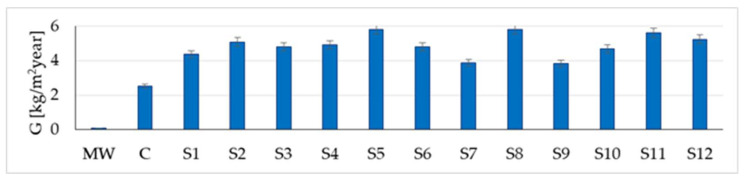
Mass (water vapour) flow G integrated over one year.

**Figure 17:**
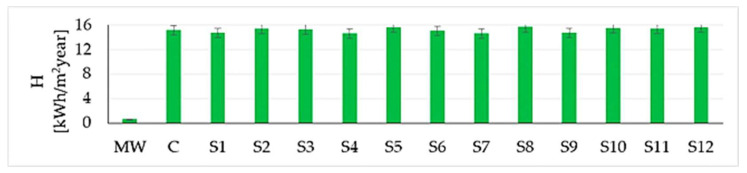
Enthalpy H calculated as the sum of the modulus integrated over one year.

Centralising the information obtained from the simulation for all the materials considered (Figure 15, Figure 16 and Figure 17), it can be said that correlations have again been identified with reports in the literature, especially in terms of mass (water vapour) flow G, integrated over one year [24]. Additionally, based on these figures, it can be said, in terms of the mineral wool core composite wall, that it shows the lowest heat flow Q (integrated over one year), almost 21 times lower than the minimum indicator for rammed earth and more than 24 times lower than the result recorded for the concrete wall. Therefore, on the one hand, the insulating quality of the mineral wool core is demonstrated, although it was not necessary, and on the other hand, it is judged that, if an interior wall is to function as a temperature buffer, it would be most convenient to use concrete or rammed earth. The moisture buffer quality of the mineral wool core composite wall is also not a strong point, with the mass (water vapour) flow indicator G (integrated over one year) again showing much lower values, more than 26 times lower than the concrete wall and more than 35 times lower than the lowest value recorded for rammed earth.

Comparing the results obtained for the 12 variants of rammed earth walls in Figure 15, Figure 16 and Figure 17, the descriptive indicators of energy/moisture exchange capacity show some variations, which are assumed to be the consequences of the density, porosity, and particle size composition specific to each type of clay soil analysed. Thus, although it is not possible to precisely identify a correlation function between these performances and the specific characteristics of the material (density, specific heat capacity, thermal conductivity, thermal diffusivity, and porosity), it can be appreciated that they have their own influence on the mass (water vapour) flow integrated over one year:

- The sample with the highest mass transfer (G = 5.82 kg/m^2^year), S5, has the highest porosity (41%), the lowest density (1490 kg/m^3^), the lowest thermal conductivity (0.523 W/mK), the lowest thermal diffusivity (3.51∙10^−7^ m^2^/s), and the highest specific heat capacity (999 J/kgK).
- The sample with the lowest mass transfer (G = 3.83 kg/m^2^year), S9, has an intermediate porosity (30%), the highest density (2150 kg/m^3^), the highest thermal conductivity (1.209 W/mK), and the second lowest specific heat (736 J/kgK) among all the tested samples.

If all this information is correlated with references in the literature [154,155], it can be appreciated that, although some of the soil samples analysed show very similar characteristics and are even placed on the ternary diagram in positions that are sometimes almost overlapping, density and porosity are elements that determine the specific hygrothermal behaviour of each type of soil; therefore, a specific analysis of each individual case is necessary. Throughout the lifetime and exploitation of a building element made of clay soil, there is a primary intervention on density and porosity characteristics at the time of installation. Subsequently, a series of changes occur at the microstructural level with the passing of the drying and maturation periods of the clay material, which continue even during the period of exploitation of the construction element (in this case, the simulated wall) when, under the influence of humidity variations, a rearrangement of the granules and a redistribution and resizing of the pores take place. As demonstrated [154], the existence of wet–dry cycles leads to a variation in the porosity distribution curve, mainly of the interconnected porosity, with drying leading to a reduction in the connectivity between pores and the number of isolated pores increasing. As a reverse effect, the wetting effect contributes to the recovery of lost pores and connections, and pore space and porosity increase after wetting. In addition, as the literature studies [154] state, hydraulic conductivity decreases due to connected porosity, the pore radius and interconnecting zone decrease, and connected macropores play a decisive role in saturated hydraulic conductivity. Thus, based on this information, it is possible to identify the importance and influence of porosity and material density (two characteristics that are continuously changing under the influence of atmospheric humidity) on the ability to function as a moisture buffer without neglecting the influence that the mineralogical composition of the particles has on these aspects [154,155,156,157].

Moreover, this hypothesis is also supported by 3D microstructural analysis [155]. At the 2D analysis level, aspects related to the variation in the degree of pore interconnection and pore dimensional variation were identified [154]. Using 3D analysis [155], these statements were again confirmed, and the relationship between the three-dimensional pore structure characteristics and the hydraulic properties was quantitatively analyzed. Thus, in the case of granite residual soil analysed by the authors, it could be observed that, by going through cycles of moisture alternation, there is a decrease in the average pore radius, in the area of connection between pores, and implicitly, in the connected porosity. In addition, it was also shown that the addition of large particles improved saturated hydraulic conductivity by increasing soil pore connectivity. Therefore, the spatial variation in the micropores and the complex pore structure help to increase water retention capacity [155]. Although the soil types analysed in the present study are different from those presented in the literature and the analysis did not assume an accumulation of liquid water in the wall following wet–dry cycles but a mass transfer originating in and from the availability of water vapour to the air in the interior space, it can be appreciated that the mechanism of functioning that allows the simulated wall to act as a moisture buffer is based on similar phenomena. The modification of porosity, and in particular of interconnected porosity, is a phenomenon that, by means of hydraulic conductivity, becomes the “activation engine” of the continuous interaction between the wall and the air in the interior space under the influence of humidity, while also continuously taking into account the clay content, sand content, particle size distribution, and mineralogical composition [158,159,160,161].

Based on the results obtained from the analysis of the simulation of the thermo-technical behaviour of walls using the WUFI^®^ 2D 4.2 software, the following can be said:

- Heat flux at the wall surface and mass flux, respectively, during the 16 years of operation showed similar evolution for all 12 types of clay material analysed, with small variations explained by differences in thermophysical characteristics specific to each type of S1–S12 earth.
- In the case of walls made from clay material, there is a stabilisation in the evolution of the moisture content phenomenon by the 5th year of simulation. This contrasts with walls made of concrete, where the characteristic moisture content appears to evolve continuously over the 16-year period. Therefore, it can be said that in the case of the construction elements of existing buildings, which have already gone through a sufficient period for the maturation of the materials in their construction elements, the rammed earth wall quickly develops a moisture buffer function. This role of moisture buffer is assumed by all the clay soil samples analysed, S1–S12, as long as their density (1490–2150 kg/m^3^) is within the limits recommended by the literature (min. 1200 kg/m^3^ [41]—max. 2200 kg/m^3^ [9]). Closely related to density, the parameter “porosity”, which, as shown, is also continuously influenced by moisture content [154,155,156,157,158,159,160,161], and which in the case of the samples analysed falls within the limits (25–44%), plays an important role in the ability of the wall to fulfil its buffer role. However, no regulation is kept indicating that high density implies low porosity or low density implies high porosity, with the density also influencing the mineralogical nature and grain size distribution of the analysed material. Similarly, it is considered that if the thermophysical characteristics of the soil samples analysed, such as specific heat capacity (701–999 J/kgK) or thermal conductivity (0.523–1.209 W/mK), factors that are involved in obtaining the role of a temperature buffer, correlate with values reported in the literature (specific heat capacity: min. 664 J/kg·K [1]—max. 1832 J/kgK [1,14] and thermal conductivity: min. 0.38 W/mK [12]—max. 2 W/mK [9]), the benefits of a rammed earth wall can be obtained. Therefore, to estimate the performance of a clay soil type material for the purpose of producing building elements that, in addition to the essential functions of strength, stability, etc., also bring benefits in terms of continuous regulation of air quality and indoor comfort, a preliminary analysis of the material is essential by experimentally determining some indicators in the laboratory and comparing them with values reported in the literature, given that this field is still insufficiently standardised.
- The comparative analysis of the behaviour of the 12 rammed earth walls and the concrete wall showed similarities in the evolution of heat and moisture transfer phenomena. However, although not analysed in this study, it is known that the concrete wall has a much more polluting impact on the environment due to the main raw material used, cement, whose production technology is a strong generator of CO_2_ and other greenhouse gases. Choosing a rammed earth wall is therefore much more eco-friendly.
- The comparative analysis conducted between the behaviour of the 12 rammed earth walls and the classic composite dry-wall system with a mineral wool core revealed significant differences. The latter cannot perform as a temperature or humidity buffer, exhibiting an enthalpy exchange with indoor air that is only 4% of that of the rammed earth walls. Consequently, it does not play a significant role in regulating indoor comfort conditions.

## 4. Conclusions

The aim of this study was to analyse the behaviour and temperature and/or moisture buffering capacity of interior rammed earth walls compared to two types of walls made of classical materials, namely concrete or mineral wool core composite, in the following variants: a compact concrete wall (concrete w/c = 0.5; density, ρ, 2300 kg/m^3^; specific heat, c_p_, 850 J/kg·K; thermal conductivity, λ, 1.60 W/m·K; porosity 0.18%; water vapor diffusion resistance factor, μ, 180) and a drywall partition system using gypsum board for pannels and a mineral wool core (WUFI^®^ 2D 4.2 database data: ρ 730 kg/m^3^; c_p_ 850 J/kg·K; λ 0.218 W/m·K; porosity 0.65%; μ 4.80) with interior mineral wool (WUFI^®^ 2D 4.2 database data: ρ 65 kg/m^3^; c_p_ 850 J/kg·K; λ 0.032 W/m·K; porosity 0.95%; μ 1.1). At the outset, it should be specified that, for compatibility of the comparisons, the dimensions of all wall variants were considered identical, but, as an interior wall made of concrete is unlikely to have a thickness as considered in the simulation, the first conclusion that can be taken into account is that this study will require further research in order to report on a concrete wall thickness closer to the usual and to identify the optimal thickness of rammed earth walls.

Based on the experimental results, it can be judged that the tested clay soil samples correspond in terms of thermophysical characteristics to materials suitable for use in the construction of the soil walls, in accordance with the specifications in the literature.

The data collected after simulating the behaviour of the walls made of these clay soil samples revealed the roles of humidity and heat buffers that such a construction element has, which can thus contribute to the regulation of air quality and indoor comfort during the entire period of use.

The scientific value and originality of this study derive from the following:

- Analysis of a large number of samples, highlighting the heterogeneity of thermophysical characteristics, and comparing these characteristics with the literature specifications;
- Evaluation of the feasibility of rammed earth walls, considering both the compatibility of the raw material (clay soil) with the intended application area and its impact on indoor air quality;
- To highlight the enthalpy evolution at the rammed earth wall level, both in warm season conditions (without the existence of other means of indoor climate control) and in cold season conditions (where a consistent indoor temperature was maintained due to additional heating). This was achieved by simulating the wall’s behaviour over an extended period (16 years) with a small tracking step (1 h), which contributed to increasing the degree of precision, accuracy, and detail of the information obtained from the simulation. The entire phenomenon is examined from the point of view of the mechanisms occurring at the wall level, identifying potential factors influencing the characteristic parameters of indoor air. Therefore, the sensible enthalpy provides information on the temperature of the wall, and the latent enthalpy provides information on its moisture content;
- Confirmation of the temperature and moisture buffering capabilities of rammed earth walls during both warm and cold periods of the year, consistent with other reports in the literature.

The main direction for further research that emerges from this study is the identification of research development possibilities, facilitated by simulation using specialised software, of the long-term behaviour of the rammed earth walls, so that easily quantifiable factors can be identified that can help establish the optimal thickness of the rammed earth walls and that, at the same time, meet the criteria of resistance and stability, as well as those of efficiency in the role of temperature and humidity buffers, and, last but not least, the criteria of economic efficiency and space organisation.

## Figures and Tables

**Table 1:** Permissible proportions, minimum/maximum limits, and clay, silt, and sand reported in the literature.

%	Alley, 1948 [49]		Houben, 1994 [50]		McHenry, 1984 [51]		Norton, 1997 [52]		Radanovic, 1996 [53]		Schrader, 1981 [54]		SAZS 724:2001 [7]	
	Min.	Max.	Min.	Max.	Min.	Max.	Min.	Max.	Min.	Max.	Min.	Max.	Min.	Max.
Clay	25	30	0	20	30	35	10	25	30	35	20	30	5	15
Silt	50	80	10	30	0	0	15	30	0	0	0	0	15	30
Sand and gravel	10	20	45	75	65	70	45	75	65	70	70	80	50	70

**Table 2:** Depth of sampling.

Sample	S1	S2	S3	S4	S5	S6	S7	S8	S9	S10	S11	S12
Depth of sampling (m)	4–4.5	2.5–3	6–6.5	8–8.5	1–1.5	2–2.5	3–3.5	9.5–10	5–5.5	2–2.5	1.5–2	1–1.5

**Table 3:** Characterisation of plasticity and consistency.

Sample	Liquidity and Plasticity Limits			Plasticity Index	Consistency Index	Characterisation
	w (%)	w_L_ (%)	w_p_ (%)	I_p_ (%)	I_c_ (-)	
S1	19.89	20.12	50.32	30.20	1.008	Brown clay, medium plasticity, hard
S2	19.68	17.24	46.21	28.97	0.916	Brownish-yellow clay with sparse calcareous concretions, medium plasticity, plastic vigorous
S3	20.34	18.80	47.90	29.10	0.947	Yellow clay, medium plasticity, plastic vigorous
S4	21.54	20.63	49.65	29.02	0.969	Grey clay, medium plasticity, plastic vigorous (brownish appearance from the CaCO_3_ content)
S5	18.22	16.59	47.17	30.58	0.947	Brownish dusty clay with macroporic aspects, medium plasticity, plastic vigorous
S6	21.66	20.34	48.47	28.13	0.953	Yellow clay, medium plasticity, plastic vigorous
S7	20.88	19.75	48.37	28.62	0.961	Yellow clay, medium plasticity, plastic vigorous
S8	19.78	17.21	45.81	28.60	0.910	Greyish dusty clay with calcareous concretions, plastic, viscous
S9	21.8	19.34	49.79	30.45	0.919	Yellow clay with greyish patches and calcareous concretions, medium plasticity, plastic vigorous
S10	20.81	18.34	48.32	29.98	0.918	Yellow clay, plastic vigorous
S11	19.99	16.24	43.10	26.86	0.860	Yellow dust clay, medium plasticity, plastic vigorous
S12	20.37	17.26	42.28	25.02	0.876	Brown, chalky plastic clay

**Table 4:** Characterisation of soils in terms of free swelling activity.

Sample	S1	S2	S3	S4	S5	S6	S7	S8	S9	S10	S11	S12
U_L_ (%)	90	75	80	82	91	78	76	82	95	92	70	90
Characterisation of soils in terms of activity	Medium activity soils											

**Table 5:** Thermophysical characteristics of the samples after conducting the oedometer test.

Sample	S1	S2	S3	S4	S5	S6	S7	S8	S9	S10	S11	S12
Density (kg/m^3^)	2073	1917	1994	1729	1490	1913	1923	1827	2150	1886	1739	1641
Thermal conductivity (W/mK)	1.146	0.882	0.930	0.679	0.523	0.931	0.988	0.552	1.209	1.040	0.716	0.839
Specific heat (J/kgK)	701	776	765	803	999	764	816	815	736	830	794	917
Porosity (%)	23.22	28.73	26.15	25.96	44.60	29.15	29.04	32.08	29.96	30.15	35.11	38.99
Eoed_200–300_ (MPa)	13.58	12.28	12.68	11.76	10.26	12.79	12.89	11.98	16.72	12.00	11.89	10.86
ε_200_ (%)	2.2	1.9	2.0	2.4	2.6	2.2	2.0	2.1	1.8	2.2	2.3	2.4
Compressibility	Medium compressibility											

## Data Availability

Not applicable.
